# The barriers and facilitators to smoking cessation experienced by women’s partners during pregnancy and the post-partum period: a systematic review of qualitative research

**DOI:** 10.1186/s12889-015-2163-x

**Published:** 2015-09-03

**Authors:** Kate Flemming, Hilary Graham, Dorothy McCaughan, Kathryn Angus, Linda Bauld

**Affiliations:** Department of Health Sciences, University of York, York, YO10 5DD UK; Institute for Social Marketing, University of Stirling, Stirling, FK9 4LA UK; UK Centre for Tobacco and Alcohol Studies, http://www.ukctas.ac.uk

**Keywords:** Pregnancy, Smoking, Partners, Qualitative research, Meta-ethnography, Systematic review

## Abstract

**Background:**

Smoking in pregnancy can cause substantial harm and, while many women quit, others continue to smoke throughout pregnancy. The role of partners is an important but relatively under-researched factor in relation to women’s smoking in pregnancy; partner’s smoking status and attitudes to smoking cessation are important influences in a pregnant women’s attempt to quit. Further understanding of how partners perceive the barriers and facilitators to smoking cessation in pregnancy is needed, particularly from qualitative studies where participants describe these issues in their own words.

**Methods:**

A synthesis of qualitative research of partners’ views of smoking in pregnancy and post-partum was conducted using meta-ethnography. Searches were undertaken from 1990 to January 2014 using terms for *partner/household, pregnancy, post-partum, smoking, qualitative* in seven electronic databases. The review was reported in accordance with the ‘Enhancing transparency in reporting the synthesis of qualitative research’ (ENTREQ) statement.

**Results:**

Nine studies reported in 14 papers were included, detailing the experience of 158 partners; the majority were interviewed during the post-partum period. Partners were all male, with a single exception. Socioeconomic measures indicated that most participants were socially disadvantaged. The synthesis identified recurring smoking-related perceptions and experiences that hindered (barriers) and encouraged (facilitators) partners to consider quitting during the woman’s pregnancy and into the post-partum period. These were represented in five lines of argument relating to: smoking being an integral part of everyday life; becoming and being a father; the couple’s relationship; perceptions of the risks of smoking; and their harm reduction and quitting strategies.

**Conclusions:**

The cluster of identified barriers and facilitators to quitting offers pointers for policy and practice. The workplace emerges as an important space for and influence on partners’ smoking habits, suggesting alternative cessation intervention locations for future parents. Conversely, health and community settings are seen to offer little support to fathers. Interventions centred on valued personal traits, like will-power and autonomy, may have particular salience. The review points, too, to the potential for health information that directly addresses perceived weaknesses in official advice, for example, around causal mechanisms and effects and around contrary evidence of healthy babies born to smokers.

**Systematic review registration:**

PROSPERO 2013: CRD42013004170

**Electronic supplementary material:**

The online version of this article (doi:10.1186/s12889-015-2163-x) contains supplementary material, which is available to authorized users.

## Background

Smoking in pregnancy can cause substantial harm and, while many women quit, others continue to smoke throughout pregnancy [1, 2]. In high-income countries, smoking in pregnancy is strongly associated with social disadvantage, in line with broader national patterns [3]. Among pregnant women in the UK, for example, prevalence is 30 and 14 % for the lowest and highest socioeconomic groups respectively; quit rates are also much higher among smokers in advantaged circumstances (72 vs 29 %) [4]. Cessation can be challenging. Some interventions are effective in promoting smoking cessation in pregnancy but their effects fade over time [5, 6]. Systematic reviews have identified a range of barriers and facilitators that pregnant women face when trying to quit smoking. These are strongly linked to social disadvantage as well as relationship factors and, as most pregnant women are in a cohabiting relationship, relationships can play a significant role [7, 8].

The role of partners is an important but relatively under-researched factor in relation to smoking in pregnancy. A partner’s smoking status and attitudes to smoking cessation are potentially important influences in a pregnant woman’s attempt to quit [9]. For example, partners who try to quit with the pregnant woman can be seen as more supportive [10] while a partner whose quit attempt fails may reduce the chances of the woman succeeding [11]. Men may be less likely to receive advice to stop from health professionals than their pregnant partners, and may be exposed to less pressure from friends and family to quit [12].

Despite these challenges, pregnancy provides an opportunity for quitting both for expectant mothers and their partners. Further understanding of how partners perceive the barriers and facilitators to smoking cessation in pregnancy is needed, particularly from qualitative studies where participants describe these issues in their own words. Their views and experiences may help inform interventions to support cessation during and after pregnancy. We therefore undertook a systematic review of qualitative studies to explore the barriers and facilitators to smoking cessation experienced by women’s partners during pregnancy and post-partum.

## Methods

### Design

A synthesis of qualitative studies of smoking partners’ views of smoking in pregnancy and post-partum was conducted using meta-ethnography [13]. Meta-ethnography is an interpretative approach to research synthesis which enables conceptual translation between different types of qualitative research [14].

### Search methods

**Fig. 1:**
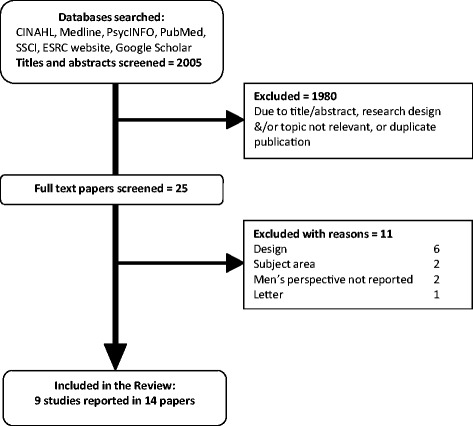
Inclusion flow diagram

We searched for published and unpublished studies from 1990 to January 2014 (Fig. 1). Terms for *partner/household, pregnancy, post-partum, smoking, qualitative* were developed by KA for searches of electronic databases (CINAHL, Medline, PsycINFO, Social Sciences Citation Index (SSCI), Economic and Social Research Council (ESRC) website, and a specific ‘ahead of print’ search in PubMed and Google Scholar) on 9-10^th^ January 2014, together with citation searching and consultation with the wider project team. Detail of the search strategy is provided in Additional file 1.

Papers from 1990 were selected for inclusion if they (a) were published in English and reported partners’ views of smoking in pregnancy and after childbirth, (b) used a qualitative research method and (c) were conducted in a higher-income country where, as in the UK, cigarette smoking is associated with social disadvantage.

### Data extraction and quality appraisal

Relevant data were extracted from papers (aim, type and number of participants, methodology used, methods of data collection, analysis, and results). Data were extracted (KF) and checked (DM) by two reviewers. Papers were appraised for quality [15] by two reviewers (KF, DM), with disagreements in scoring resolved by consensus. Quality scores ranged from 23–32 (Additional file 2). The thesis by Gage [16] was not quality appraised, as the checklist used was not designed for theses. The journal paper arising from the thesis [17] was however quality appraised. There was no *a priori* quality threshold for excluding papers; assessment was undertaken to ensure transparency in the process.

### Synthesis

Meta-ethnography has four iterative phases (Table 1). For Phase 1, three reviewers (KF, HG, DM) read all papers in depth. Phase 2 involved line-by-line coding of data (participant accounts as reported in the primary papers and authors’ interpretations) in each paper (KF) relating to men’s perceptions of barriers and facilitators to quitting, using ATLAS.ti Software [18].

**Table 1:** Phases of meta-ethnography (adapted from Noblit and Hare [13]) [14]

Phase of meta-ethnography	Processes involved
Phase 1 Reading the studies	Developing an understanding of each study’s context and findings.
Phase 2 Determining how the studies are related	Comparing contexts and findings across and between studies.
Phase 3 Translating the studies into one another	Mapping similarities and differences in findings and translating them into one another; the translations represent a reduced account of all studies. (First level of synthesis)
Phase 4 Synthesising translations	Identifying translations that encompass each other and can be further synthesised; expressed as ‘lines of argument’. (Second level of synthesis)

The codes were compared and grouped by the reviewers (KF, DM with HG) into broad areas of similarity through reciprocal translation analysis (RTA) (Phase 3) to generate a reduced set of codes (translations) about barriers and facilitors that partners perceive related to smoking cessation. Phase 4 focused on these translations. The reviewers examined and compared them to identify five ‘lines of argument’. These capture recurring smoking-related perceptions and experiences that hindered (barriers) and encouraged (facilitators) partners to consider quitting during the woman’s pregnancy and into the post-partum period.

## Results

### Results of searching and study characteristics

Of 2005 potentially-relevant papers, 1991 were excluded. Nine studies reported in 14 papers were included in the review (Figure 1, Additional file 2). The studies reported the experiences of 158 partners, aged 16–59 years. Partners were all reported as male, with a single exception [19]. The perspectives of this same-sex partner were not separately identified and the researchers described all participants using the masculine pronoun. It is therefore primarily the perspectives of male partners and fathers that inform our review. Of the participants, 93 were interviewed in the post-partum period (up to six months), 49 were interviewed during their partner’s pregnancy, whilst the timing of interview was unclear for the remaining 16 interviewees. Five studies were conducted in Canada, two in Australia and one each in the UK and the USA. Of the 14 papers, one was published in 1998, seven between 2000 and 2009 and six since 2010.

Socioeconomic measures (employment status, educational level, occupational group) indicated that most study participants were socially disadvantaged. Other participant characteristics were inconsistently reported, including partners’ smoking status. However, studies reported that most were current or recent ex-smokers; only eleven partners across four studies were described as non-smokers.

### Identification of lines of argument

The synthesis identified five recurring themes (lines of argument) running through partners’ perceptions and experiences of smoking. These related to smoking being an integral part of everyday life; becoming and being a father; the couple’s relationship; perceptions of the risks of smoking; and their harm reduction and quitting strategies.

### Smoking as part of everyday life

Smokers reported that barriers to quitting were built into their domestic, social and working lives. Smoking was integral to all these spheres, with participants acknowledging that they could not imagine their lives without cigarettes [20–23]. Domestically, smoking was a shared activity, part of a couple’s life together and part of their wider family circle [21, 22].

*‘Family members more or less not so much encourage you to smoke, but they prefer you to keep smoking.’* [22]

Workplaces were often further barriers to quitting, particularly where smoking was the norm [23]. They afforded fathers the freedom to smoke without surveillance from, or risk to, their child or partner. Smoking could therefore be kept separate from family life and domestic responsibility [24]. Conversely, workplaces where smoking was not the norm were seen to facilitate cutting down [23].

*‘The* [supervisor] *gives two packs so everyone can smoke*…*I can’t* [quit] *because in the working area everyone is smoking…including the boss.’* [23]

At home and at work, being a smoker was seen to convey autonomy and independence and assert a positive masculine identity [20, 24]. Men felt they had a ‘right’ to smoke which was curtailed by tobacco control measures banning smoking in public places [12]. Further barriers to quitting lay in smoking being a source of enjoyment and an addiction from which it was difficult to escape [16, 20–25].

*‘I guess you can say, just who doesn’t want to sit on their couch and have a smoke?’* [23]

*‘… I’m addicted to the craving of the nicotine. There’s no way you could stop me unless you cut off my hand or cut off my mouth and probably then I won’t smoke.’* [21]

In addition, study participants spoke of smoking as a way to maintain emotional stability and manage stress. It enabled them to be caring partners and fathers, providing both a mood-management strategy and a ‘time out’ [16, 20, 24–26]. Some study participants cited stresses around their partner’s pregnancy, including her changes in mood, with smoking described as *‘…the excuse to get out of the house*’ [25].

*‘I need to relax myself… from the hard work. I* [do] *not really want to quit it* [smoking]*. I need to enjoy my life and I’m not making any trouble to my family.’* [20]

*‘I don’t think it has anything directly to do with cigarette smoking curing your stress or anything, I think it is more of a break from everything, to go ignore everything....let yourself re-focus your thoughts.’* [16]

*‘…it gives me that two-three minutes in another zone.’* [25]

In the workplace, too, smoking was regarded as a stress management intervention, for workers and supervisors, as well as collectively being a signal for a pause in the working day [23].

### Becoming and being a father

Parenthood was experienced as a life transition with the potential to facilitate changes in smoking behaviour [16, 17, 20, 21, 25–27]. Fathers spoke of their feelings towards the baby, both during their partner’s pregnancy and in the months after birth, and that being a smoker was at odds with the person they wanted to be [16, 17, 20, 21, 25–27]. Those unable to quit in pregnancy anticipated that the baby’s birth would enable them to succeed [27].

The key motivation was the perception that smoking was incompatible with being a ‘good father’ [16, 17, 20, 24–26]; a role model who put their children’s needs first and wanted ‘*to live to see all your kids grow up’* [21]. Parenthood therefore unsettled the taken-for-granted place that smoking had in their lives [17, 20, 25–27].

*‘I don't want to get lung cancer and die so, and leave the little kid without a dad. …and, well I mean there's a lot more expenses having a baby …that's really where the money should be going…’* [26]

*‘Because Daddy does it and you soon learn that by all means your kids look up to you and I just, I don’t want him to have that impression. So if anything actually was to make me think more about quitting now, it would be that reason.’* [27]

*‘If my kids will say ‘Daddy don’t do that* [smoke]*’, I will* [quit]*.’* [25]

*My son, he needs me. If you’d tell me to quit, I’d probably quit for him.’* [25]

While parental responsibilities could facilitate quitting, they could work against it [17, 20, 25–27].

*‘I had to graduate…look for a new job....finding out my wife was pregnant…and all those things right and moving and so on there was quite a few stress*[ors]*, but eventually I suppose what really made me quit was the baby.’* [21]

*‘You have to prepare for the baby, you have to buy things, you have to do everything….I just started smoking more.’* [25]

The study by Gage [16, 17] explored in depth how becoming a father impacted on smoking behaviour. Through pregnancy, the men’s quit plans were continually revised. Men who did not manage to stop re-focussed on harm reduction. They spoke of how they protected their pregnant partner and, after birth, the new baby, by not smoking in proximity to them, a strategy that enabled them to maintain their ‘good father’ identity.

*‘Yeah I smoked a lot, but I cut down a whole lot. I smoked at least double what I smoke now. I only smoke when I am at work or I go outside.’* [17]

*‘During the pregnancy, what I thought is I am not smoking in front of her. I’m just keeping her away whenever I smoke so that she cannot breathe the smoke. I’ll go outside to smoke…’* [25]

Smoking alone was seen to protect the baby and other children and, thereby, to demonstrate their ‘good father’ qualities [23], in effect separating the identities of smoker and father [20].

*‘I’ll go for hours with them* [infants] *awake where I don’t smoke, but if they go down for a nap and they both go down together I’ll sit and smoke one right after the other to make up for it.’* [20]

Some men spoke of their concerns about the smell from second hand smoke (SHS) when they smoked near their partners and from third hand smoke (THS) from clothes and furnishings [25]. They described how they brushed their teeth, used gum, and washed their hands and face after smoking [25, 26]. For those hiding their smoking, concealment became an additional reason for these body-hygiene practices.

*‘Even though I quit smoking, she still knows that I’m smoking, or I’m having occasional cigarettes. And even with multiple attempts to try and mask the smell, she can still smell it. So I can’t really hide it…’* [25]

Post-delivery, the men in Gage’s study [16, 17 ] described their reduced motivation to quit, a time when social pressures to quit were also reduced. Study participants noted that being a father was their main priority and the baby was too young to be influenced by their behaviour. However men recognised both the general stigma of being a smoker and the specific stigma directed at parents who smoke [12, 20, 26]. This latter source of stigma could trigger guilt and shame that they were failing to be a ‘good father’.

*‘*[Smoking] *wasn’t as pleasurable, due to the guilt of knowing what I was doing to myself because now I’m a father and I’ve got someone to take care of.’* [26]

### The couple’s relationship

Most partners were current or recent ex-smokers and many were part of a smoking couple. The sub-sections below look at this group first before turning to partners living with a non-smoker and the small minority of partners who were non-smokers.

#### Partners in smoking couples

Across the studies, it was clear that smoking was a significant part of their relationship. As noted above (in the section on smoking as part of everyday life), smoking was a shared and bonding activity. This common bond could facilitate a shared approach to quitting; the domestic cues for smoking were removed and partners provided mutual support [25, 27].

*‘I want my girlfriend to give up, but why should she give up if I don’t.’* [12]

*‘As a partner I think any partner should respect what the pregnant woman is going through and if she’s not allowed to do anything, it’s just easier and you actually feel as if you are contributing if you stop doing something.’* [27]

*Father: ‘Well there weren’t much people to give me influence that helped. The wife’s not bringing any cigarettes into the house. That’s a big bonus.’*

*Mother: ‘He was getting cravings too. But again, he wouldn’t light up because it was me. Because he knew that it was just going to torture me even more.’* [27]

However, it was clear that many partners found it hard to make and deliver on commitments to quit together [12, 16, 19, 24]. Thus, despite an initial agreement, the pregnant woman often found herself reducing or quitting on her own [12, 16, 19, 24]. A barrier was the value partners placed on independence and autonomy; being ‘pushed’ into agreements to quit was seen to compromise core elements of their masculinity and create tension and arguments [16, 24]. In addition, some partners considered that joint quitting would leave them unable to support the pregnant woman as she went through the stress of quitting [12, 20].

*‘If my wife decided tomorrow to stop, I don't know if tomorrow I would be prepared to stop. In my mind, I know I want to stop, but I don't know if physically I can stop tomorrow.’* [12]

*‘I think it is better that we just do what we feel is more comfortable for us, for each individual one of us at the time.... so that we don’t affect the other person in a positive or negative way.’* [16]

*‘Generally just pushing me and pushing me to quit would end up in an argument.’* [24]

*‘One stressed out person in the home was bad enough, without there being two.’* [12]

Thus, rather than quitting together, study participants were more likely to describe supporting their pregnant partner to change her smoking behaviour. This support-giving ranged across a continuum, from positive and enabling to negative and controlling.

Examples of facilitative support-giving were undertaking activities together and praising the pregnant woman when she cut down on smoking [12, 16, 27]. Support-giving could also include recognising the effect of their own smoking habits and modifying where and when they smoked [12, 17, 20, 27]. However, some partners denied being a smoker was an influence, particularly when there was smoking-related tension in the relationship [28].

*‘I am trying to be supportive and not smoking around her, but when I do and she sees me having one, it’s all the more harder* [for her]*.’* [27]

*‘I think that* [my smoking] *maybe used to tick her off a little bit too cause, but you know like I was kind of inconsiderate…’* [20]

There were some instances where support-giving included support for their pregnant partners continuing to smoke because *‘the stress of going without cigarettes would cause the baby more harm than smoking’* [12].

More controlling behaviours included ‘policing’ the woman’s behaviour by stipulating times and places where smoking was permitted, confiscating her cigarettes, restricting her access to money and threatening to disclose her continued smoking to disapproving family members. Study authors noted that, anchored in male privilege and economic power, these behaviours increased anxiety and guilt for the woman [19, 27, 28].

#### Smoking partners living with non-smoking pregnant women

Study participants in this group spoke of being held accountable for their smoking behaviour, with rules imposed to which they were expected to conform. They described their pregnant partner’s disapproval and pressure to quit [12, 24]. In response, some smoked covertly, including in the workplace (see section above on smoking as part of everyday life) [23, 24].

*‘If I see her coming or whatever I put it out....it’s one thing that she knows that I smoke – I think that it’s another thing that she sees me smoking.’* [24]

There was a perception that encouragement to quit by their pregnant partner compromised their independence and autonomy, and therefore became both a source of tension and resentment in their relationships and a barrier to quitting [12, 20, 21, 24]. Like those in a smoking couple, self-determination over quitting was perceived to the key to success.

*‘My wife never really pushed me to quit, I told her I’ll quit on my own terms....’* [24]

#### Non-smoking partners living with smoking pregnant women

Less than 10 % of study participants were non-smokers, and their relationship with their smoking partner, during and after her pregnancy, was the only area in which they were separately identified. This group reported that pregnancy positioned the place of smoking in the couple’s relationship under scrutiny, and it was challenging to know how best to respond [19, 28]. In relationships where there could be an open dialogue about their partner’s smoking, men were more understanding of the difficulties of quitting [16]. It was acknowledged that exerting pressure to quit could result in increased levels of smoking [16]. There was an acceptance that resumption to smoking post-partum was likely and that this would be accepted [19].

*‘I'd probably accept it…I know how strong an addiction is, and so to keep the peace…I don't really want her sneaking around me…and then having this whole…mistrust. So I'll probably just accept her decision* [to smoke]*.’* [19]

### Risk perceptions

Risk perceptions of maternal smoking in pregnancy and of foetal and child exposure to second hand smoke (SHS) have the potential to facilitate quitting. However, the review data suggest that this appraisal more commonly acted as a barrier to quitting.

The major factor was scepticism about the scientific evidence. While study participants were aware that smoking in pregnancy posed risks to the unborn child, they spoke of a ‘lack of hard proof and hard facts’ [12]. What was missing was information on how and in what ways smoking damaged the unborn child and people’s health more generally [12, 17, 27]. A common view was that, without incontrovertible evidence, there was little motivation to change one’s smoking behaviour.

*‘I don’t know if they have proved it* [smoking related pregnancy risks]*, I mean there is a lot of scare-mongering with children.’* [29]

*‘We're already getting the messages, but we’re not getting the facts and figures. The warnings are there, but there’s nothing backing it up.’* [12]

*‘It would have to be more concrete to hit me hard. To just say something to me, it’s almost like coming up to me in a restaurant…in the smoking area…and have someone come up to you and ask you to quit smoking....it would have to be something more concrete for sure, for it to have any impact.’* [27]

Partners reflected, too, on what they saw as inconsistencies between government advice on the dangers of smoking and its tobacco control policies [17, 22, 29].

*‘I have just one big gripe with the government…because they’re the ones and organisations are pushing all this non-smoking, stop smoking and all the rest of it, but if they were really serious about it, stop selling them.’* [22]

Personal experience fed into the scepticism about the risks of smoking. Partners noted that their knowledge of healthy babies born to maternal and paternal smokers was at odds with ‘official’ advice, and reduced their motivation to quit [12, 16, 17, 22, 29].

*‘I myself, I don’t think it has any effect. I come from a family of six kids and my father smoked right through the whole of us…it didn’t do anything to us.’* [12]

*‘She has got four kids and she smoked for all of her kids and look at all her kids, they are all big, bulky and healthy.’* [22]

First-time parents tended to be more concerned about the risks of parental smoking, becoming more relaxed with subsequent pregnancies as their children appeared to be healthy [12, 16]. However, despite a general scepticism about the dangers of smoking, it was widely considered inappropriate to smoke in the presence of pregnant women and young children [12, 17, 20, 22, 25]. However, the risks of SHS exposure were primarily seen to be to the baby after birth.

*‘I wouldn’t smoke in a room with a pregnant woman unless she said it was alright. It’s annoying and irritating, but it’s not going to kill anyone.’* [12]

*‘…my wife can leave the room if she wants to…he* [new baby] *can’t do anything.’* [25]

Both smokers and non-smokers shared the perception that babies were more at risk from SHS after than before birth. Some partners noted, too, that, *in utero*, the pregnant woman shielded the child from harm [12, 20]. Protecting babies was therefore the priority, with rules established for the home for the couple and for visitors [17, 22, 25].

*‘Yeah, it is protecting my child from growing up in a house with cigarette smoke. I am going to put a little awning out back for people to go under, or they can go out here on the front porch and smoke. I want to make the house and the baby smoke free.’* [17]

Smoke-free areas enabled partners to continue smoking; however, restricting one’s own smoking to these areas could be challenging.

*‘I feel guilty all of the time when I, you know, have a cigarette and I’ll come in the house and the telephone will ring or something, and she’ll* [partner] *will just hand me the baby and I know I’ve got smoke on my breath and the baby wants attention so I’ve got to talk to him and remember not to breathe into his face.’* [20]

Some partners considered that SHS exposure was a risk only for young babies and young children; a smoke-free environment was therefore only required at this stage [20].

*‘Oh as far as the baby’s concerned? Just keep the smoke away from the baby…By the time it becomes six months old, if we take* [the baby] *over and someone happens to be smoking in somebody’s house, the kid’s not gonna be that much worse for wear.’* [20]

The studies also provided some evidence on risk perceptions around third hand smoke. Again, the dangers were seen as primarily to babies.

*‘If you think about it by the time it’s been in my lungs and into the atmosphere and then into someone else’s lungs there is not much chemicals left.....plus it’s altered in the mother’s lungs too so the baby wouldn’t get anything.’* [12]

*‘Ok, you breathe them in and breathe them out* [chemicals]*, and they’re gone But in reality, they still linger, they’re still in you and you breathe them out onto the baby, onto the child….when you go to the newborn, you know that they’re fragile and they’re spotless – clean inside and out – you may contaminate them.’* [25]

### Partners’ strategies for quitting

The studies describe a range of strategies to support quitting. This included avoiding smoking cues (for example, not socialising with smokers), avoiding drinking alcohol, setting targets and physical exercise, as well as gradual reduction and the use of nicotine replacement therapy [21, 24, 25]. However, abrupt quitting was highly valued, with some partners noting that it was consistent with valued attributes of decisiveness, autonomy and will-power [21, 25].

*‘Once I put my mind to something, I’m very focussed on it…if I want something I am going to get it…I’m going to do it.’* [25]

Abrupt quitting was perceived as an event, rather than a process, and the use of cessation aids, including nicotine replacement therapy, could signal weakness and an inability to handle withdrawal symptoms [21]. An unsuccessful attempt was often described as starting smoking again rather than failing to quit [21].

Quit dates were often set for the future and then postponed as they were reached. This perpetual deferment was acknowledged as a way of managing partner expectations; it simultaneously signalled that quitting was important and delayed it [21, 24].

*‘We made these arrangements many times, so like I said, I’ll quit before you move in,* [then] *I’ll quit two months or a month before the baby is born so I’m done with all of the withdrawal and stuff by the time the baby is born......So we make these negotiations and then they always just kind of… they kind of just disappear.’* [21]

*‘She came out and said you have to quit before the baby comes and I said yeah, I know....and then ignored it and went onto something else…I never gave her the solid answer yes.’* [24]

Those making plans to quit appeared to do so without input from health professionals or cessation advisors [21, 25]. Like other partners, they noted that, while the smoking status of the pregnant woman was discussed at clinic appointments, their own smoking status was rarely mentioned. Where it was, partners considered that it was not followed up with advice or support [12, 16].

*‘What gets me is…if it’s so bad…they ask you if you smoke and that’s the end of it. They don't ask do you want some help stopping?’* [12]

## Discussion and conclusions

To our knowledge, this is the first systematic review of qualitative studies reporting partners’ perceptions and experiences of smoking cessation during and after pregnancy. Using extensive searches from 1990, we identified nine studies (14 papers) representing approximately 150 participants. While searching non-English journals may have increased the pool of studies, our review points to a major evidence gap.

The small number of studies is also a limitation of our review. With only one same-sex couple, the smoking-related experiences of gay and lesbian parents have yet to be captured. Further, five of the nine studies were Canadian, three of which were conducted by the same research group, and the one UK study included only five men. Nonetheless, our review uncovered recurrent perceptions and experiences running across place and time, suggesting that the findings can be generalised to the wider population.

A second potential limitation relates to the methods of qualitative synthesis. These are still being refined [30, 31] and can lack transparency [32]. We therefore used an established methodology for coding and synthesis. In addition, computer software (ATLAS.ti) provided ‘an audit trail’ of the interpretative process in line with the ‘Enhancing Transparency in Reporting the Synthesis of Qualitative Research’ (ENTREQ) guidance [31]. A further limitation surrounding the use of meta-ethnography and other methods of qualitative synthesis is the difficulty of accessing what Schutz [33] describes as first order constructs i.e., the full set of participants’ accounts. As reviewers we can only work with the data provided in the papers and therefore the findings of any review cannot represent the entirety of the data set to which the paper authors have access [32].

The review has been informed by studies in which the majority of partners were experiencing social disadvantage. In high income countries such as the UK, there are strong social gradients in smoking uptake and quitting [34]. Without evidence from partners in advantaged circumstances, it is not possible to draw conclusions about the specific barriers and facilitators to quitting that they experience. However, the broad lines of argument identified through our review – for example, around the importance of workplace cultures and practices, being ‘a good father’ and the quality of the couple’s relationship – are unlikely to be class-specific.

While mindful of these limitations, some broad conclusions can be drawn from our review. It identified a cluster of related barriers to quitting in the lives of men who smoke. This includes the place of smoking in domestic and working lives that are often experienced as stressful as well as scepticism about official advice contradicted by their experience of apparently healthy babies being born to smokers and into smoking families. It includes, too, investment in forms of masculinity that required self-directed and independent quitting without cessation aids. As such, smoking cessation interventions for fathers may need to reframe the focus on abrupt quitting. In addition to noting its benefits for the smoker and their family, advice and information on abrupt quitting could be provided in ways that align it with valued psychological attributes, such as will-power and autonomy.

Our review also identified facilitators to quitting. Central here is the commitment to being a good father and supportive partner. As for pregnant women [8], the transition to parenthood challenges long-established habits and motivates positive change. It therefore opens up opportunities for couples in equal and supportive relationships to quit together. The time-window for quitting is not restricted to pregnancy and the post-partum period; it is clear that children are important agents in the change process, with anticipated pressure from them identified as providing the strongest motivation for men to quit.

There are points through pregnancy and the post-partum period that may find partners more responsive to interventions to support quitting. At the time at which pregnancy was confirmed, partners can have a heightened awareness that being a smoker is in conflict with being a ‘good father’. Where quit attempts were not attempted or were unsuccessful at the start of pregnancy, concerns over being a smoker appeared to peak again in the immediate post-partum period. Targeting cessation interventions for fathers at these two key time-points may be an effective way of enhancing cessation support for this group.

The review offers pointers for policy and practice. The workplace emerges as an important space for and influence on partners’ smoking habits. The studies point to the workplace as a barrier to quitting – for example, workplace cultures where smoking affirms group membership and a masculine identity and workplace stressors seen to militate against quitting. However, workplaces could also facilitate quitting, for example, by stronger pro-family policies and practices [35], including parent-designed workplace interventions [36]. Evidence from our review that healthcare and community settings are seen to offer little support to (future) fathers also indicates the potential for paternal smoking cessation to be part of workplace programmes. Workplace interventions centred on valued personal traits, like will-power and autonomy, may have particular salience. The review points, too, to the potential for health information that directly addresses perceived weaknesses in official advice, for example, around causal mechanisms and effects and around contrary evidence of healthy babies born to smokers. Such information may be particularly relevant for partners who see their primary role as supporting their pregnant partner to quit. This group includes both partners who quit at the same time as the pregnant woman and those who continue to smoke and try and minimise harm through reducing exposure to SHS.

Given the limited pool of studies, these pointers require testing and refining through further research. Crucially the extent of qualitative research which addresses partners’ perceptions of smoking in pregnancy is limited to a small number of studies undertaken in only four countries. Further in-depth qualitative research undertaken with larger populations and across settings will add a much required depth of perspective to this field. Nonetheless, the findings of this review indicate the capacity of qualitative studies to harness lay perspectives and thereby enrich the evidence base for policy.

## Additional files

Additional file 1:
**Contains the full search strategy used for the systematic review.** (PDF 85 kb) Additional file 2:
**Contains details of the included papers, grouped by study.** (PDF 288 kb)
