# Supplementary material for: The barriers and facilitators to smoking cessation experienced by women’s partners during pregnancy and the post-partum period: a systematic review of qualitative research
Source: BMC Public Health. 2015 Sep 3;15:849. doi: 10.1186/s12889-015-2163-x (PMC4558795; doi:10.1186/s12889-015-2163-x)
Supplement: Additional file 1: — Contains the full search strategy used for the systematic review. (PDF 85 kb) [file 12889_2015_2163_MOESM1_ESM.pdf]

Additional File 1: Search strategy for *"The barriers and facilitators to smoking cessation experienced by women's partners during pregnancy and the post-partum period: a systematic review of qualitative research"*

PsycINFO
Searched via EBSCO interface, 09.01.14
Limiters - Published Date: 19900101-20140131; English

#	Query
S72	S71
S71	S4 AND S10 AND S22 AND S70
S70	S23 OR S24 OR S25 OR S26 OR S27 OR S28 OR S29 OR S30 OR S31 OR S32 OR S33 OR S34 OR S35 OR S36 OR S37 OR S38 OR S39 OR S40 OR S41 OR S42 OR S43 OR S44 OR S45 OR S46 OR S47 OR S48 OR S49 OR S50 OR S51 OR S52 OR S53 OR S54 OR S55 OR S56 OR S57 OR S58 OR S59 OR S60 OR S61 OR S62 OR S63 OR S64 OR S65 OR S66 OR S67 OR S68 OR S69
S69	(TI (acquaintance*) OR AB (acquaintance*))
S68	(TI (friend*) OR AB (friend*))
S67	DE "Friendship"
S66	(TI (visitor*) OR AB (visitor*))
S65	(TI (guest*) OR AB (guest*))
S64	(TI (flatmate* OR flat-mate* OR co-renter* OR corenter*OR roommate OR roommates OR room-mate OR room-mates OR lodger* OR tenant* OR landlord* OR land-lord*) OR AB (flatmate* OR flat-mate* OR co-renter* OR corenter*OR roommate* OR room-mate* OR lodger* OR tenant* OR landlord* OR land-lord*))
S63	DE "Roommates"
S62	(TI ((flat* OR apartment* OR room OR rooms OR house* OR home* OR accommodation) N1 shar*) OR AB ((flat* OR apartment* OR room OR rooms OR house* OR home* OR accommodation) N1 shar*))
S61	(TI (cohabit* OR co-habit*) OR AB (cohabit* OR co-habit*))
S60	DE "Cohabitation"
S59	(TI (household*) OR AB (household*))
S58	DE "Home Environment"
S57	DE "Living Arrangements"
S56	DE "Housing"
S55	(TI (relative#) OR AB (relative#))
S54	(TI (child OR children*) OR AB (child OR children*))
S53	(TI (son OR sons OR daughter*) OR AB (son OR sons OR daughter*))
S52	(DE "Family Members" OR DE "Adopted Children" OR DE "Adult Offspring" OR DE "Ancestors" OR DE "Biological Family" OR DE "Cousins" OR DE "Daughters" OR DE "Foster Children" OR DE "Grandchildren" OR DE "Grandparents" OR DE "Illegitimate Children" OR DE "Inlaws" OR DE "Orphans" OR DE "Sons")
S51	(TI ((in OR common) N1 (law OR laws)) OR AB ((in OR common) N1 (law OR laws)))
S50	(TI (grand*) OR AB (grand*))
S49	(TI (mother#) OR AB (mother#))
S48	DE "Mothers"
S47	(TI (step-*) OR AB (step-*))
S46	(DE "Stepfamily" OR DE "Stepparents" OR DE "Stepchildren")
S45	DE "Family Relations"
S44	DE "Interpersonal Interaction"
S43	(TI (family OR families OR familial) OR AB (family OR families OR familial))
S42	DE "Family"
S41	DE "Extended Family"
S40	(TI (sibling*) OR AB (sibling*))
S39	DE "Siblings"
S38	DE "Parents"

S37	(TI (significant N1 other*) OR AB (significant N1 other*))
S36	DE "Significant Others"
S35	(TI (partner*) OR AB (partner*))
S34	(TI (wife OR wives) OR AB (wife OR wives))
S33	(TI (husband*) OR AB (husband*))
S32	(TI (spous*) OR AB (spous*))
S31	((DE "Spouses" OR DE "Husbands" OR DE "Wives"))
S30	DE "Couples"
S29	(TI (married OR marriage*) OR AB (married OR marriage*))
S28	DE "Marriage"
S27	DE "Marital Relations"
S26	DE "Father Child Relations"
S25	(TI (father*) OR AB (father*))
S24	DE "Fathers" OR DE "Adolescent Fathers" OR DE "Single Fathers"
S23	DE "Expectant Fathers"
S22	S11 OR S12 OR S13 OR S14 OR S15 OR S16 OR S17 OR S18 OR S19 OR S20 OR S21
S21	TI "expectant mother*" OR AB "expectant mother*"
S20	TI (pregnant or pregnanc*) OR AB (pregnant or pregnanc*)
S19	DE "Breast Feeding"
S18	DE "Postnatal Period"
S17	DE "Prenatal Care" OR DE "Childbirth Training"
S16	DE "Perinatal Period"
S15	DE "Prenatal Diagnosis"
S14	DE "Prenatal Exposure"
S13	DE "Fetus"
S12	DE "Obstetrical Complications"
S11	DE "Pregnancy" OR DE "Adolescent Pregnancy"
S10	S5 OR S6 OR S7 OR S8 OR S9
S9	DE "Passive smoking"
S8	DE "Smokeless Tobacco"
S7	DE "Nicotine"
S6	DE "Smoking Cessation"
S5	DE "Tobacco Smoking"
S4	S1 OR S2 OR S3
S3	qualitative
S2	interview* or (DE "Interviews") or (DE "Interviewing")
S1	findings

Medline
Searched via EBSCO interface, 09.01.04
Limiters - Date of Publication: 19900101-20140131; English Language

#	Query
S67	S66
S66	S4 AND S14 AND S26 AND S65
S65	S27 OR S28 OR S29 OR S30 OR S31 OR S32 OR S33 OR S34 OR S35 OR S36 OR S37 OR S38 OR S39 OR S40 OR S41 OR S42 OR S43 OR S44 OR S45 OR S46 OR S47 OR S48 OR S49 OR S50 OR S51 OR S52 OR S53 OR S54 OR S55 OR

	S56 OR S57 OR S58 OR S59 OR S60 OR S61 OR S62 OR S63 OR S64
S64	TI (acquaintance*) OR AB (acquaintance*)
S63	TI (friend*) OR AB (friend*)
S62	MH "Friends"
S61	TI (visitor*) OR AB (visitor*)
S60	TI (guest*) OR AB (guest*)
S59	TI (flatmate* OR flat-mate* OR co-renter* OR corenter*OR roommate OR roommates OR room-mate OR room-mates OR lodger* OR tenant* OR landlord* OR land-lord*) OR AB (flatmate* OR flat-mate* OR co-renter* OR corenter*OR roommate* OR room-mate* OR lodger* OR tenant* OR landlord* OR land-lord*)
S58	TI ((flat* OR apartment* OR room OR rooms OR house* OR home* OR accommodation) N1 shar*) OR AB ((flat* OR apartment* OR room OR rooms OR house* OR home* OR accommodation) N1 shar*)
S57	TI (cohabit* OR co-habit*) OR AB (cohabit* OR co-habit*)
S56	TI (household*) OR AB (household*)
S55	MH "Residence Characteristics"
S54	MH "Housing"
S53	TI (relative#) OR AB (relative#)
S52	TI (child OR children) OR AB (child OR children)
S51	TI (son OR sons OR daughter*) OR AB (son OR sons OR daughter*)
S50	TI ((in OR common) N1 (law OR laws)) OR AB ((in OR common) N1 (law OR laws))
S49	TI (grand*) OR AB (grand*)
S48	TI (mother#) OR AB (mother#)
S47	DE "Mothers"
S46	TI (step-*) OR AB (step-*)
S45	MH "Family Relations"
S44	MH "Interpersonal Relations"
S43	TI (family OR families OR familial) OR AB (family OR families OR familial)
S42	MH "Family"
S41	TI (sibling*) OR AB (sibling*)
S40	MH "Siblings"
S39	MH "Parents"
S38	TI (significant N1 other*) OR AB (significant N1 other*)
S37	TI (partner#) OR AB (partner#)
S36	TI (wife OR wives) OR AB (wife OR wives)
S35	TI (husband*) OR AB (husband*)
S34	TI (spous*) OR AB (spous*)
S33	MH "Spouses"

S32	TI (married OR marriage*) OR AB (married OR marriage*)
S31	MH "Marriage"
S30	MH "Paternal Behavior"
S29	MH "Father-Child Relations"
S28	TI (father*) OR AB (father*)
S27	MH "Fathers"
S26	S15 OR S16 OR S17 OR S18 OR S19 OR S20 OR S21 OR S22 OR S23 OR S24 OR S25
S25	TI "expectant mother*" OR AB "expectant mother*"
S24	MH "Pregnant Women"
S23	TI (pregnant or pregnanc*) OR AB (pregnant or pregnanc*)
S22	MH "Maternal Health Services+"
S21	MH "Breast Feeding"
S20	MH "Postpartum Period+"
S19	MH "Prenatal Care"
S18	MH "Perinatal Care"
S17	MH "Prenatal Diagnosis+"
S16	MH "Infant, Newborn"
S15	MH "Pregnancy+"
S14	S5 OR S6 OR S7 OR S8 OR S9 OR S10 OR S11 OR S12 OR S13
S13	MH "Tobacco Smoke Pollution"
S12	MH "Tobacco, Smokeless"
S11	MH "Nicotine"
S10	MH "Smoking Cessation"
S9	MH "Tobacco Use Cessation"
S8	MH "Tobacco Use Disorder"
S7	TI ((stop* or quit* or reduc* or give up or giving up) N2 (cigarette* or tobacco or smoking)) OR AB ((stop* or quit* or reduc* or give up or giving up) N2 (cigarette* or tobacco or smoking))
S6	MH "Tobacco"
S5	MH "Smoking"
S4	S1 OR S2 OR S3
S3	qualitative
S2	interview* or MH "Interview"
S1	findings

CINAHL
Searched via EBSCO interface, 09.01.14
Limiters - Published Date: 19900101-20140131; English Language

#	Query
S75	S74
S74	S4 AND S11 AND S28 AND S73
S73	S29 OR S30 OR S31 OR S32 OR S33 OR S34 OR S35 OR S36 OR S37 OR S38 OR S39 OR S40 OR S41 OR S42 OR S43 OR S44 OR S45 OR S46 OR S47 OR S48 OR S49 OR S50 OR S51 OR S52 OR S53 OR S54 OR S55 OR S56 OR S57 OR S58 OR S59 OR S60 OR S61 OR S62 OR S63 OR S64 OR S65 OR S66 OR S67 OR S68 OR S69 OR S70 OR S71 OR S72
S72	TI ((live OR living OR dwell* OR reside*OR stay* OR lodg* OR inhabit*) N1 together) OR AB (live OR living OR dwell* OR reside*OR stay* OR lodg* OR inhabit*) N1 together
S71	TI (common N1 abode*) OR AB (common N1 abode*)
S70	TI (acquaintance*) OR AB (acquaintance*)
S69	TI (friend*) OR AB (friend*)
S68	TI (visitor*) OR AB (visitor*)
S67	TI (guest*) OR AB (guest*)
S66	TI (flatmate* OR flat-mate* OR co-renter* OR corenter*OR roommate OR roommates OR room-mate OR room-mates OR lodger* OR tenant* OR landlord* OR land-lord*) OR AB (flatmate* OR flat-mate* OR co-renter* OR corenter*OR roommate* OR room-mate* OR lodger* OR tenant* OR landlord* OR land-lord*)
S65	TI ((flat* OR apartment* OR room OR rooms OR house* OR home* OR accommodation) N1 shar*) OR AB ((flat* OR apartment* OR room OR rooms OR house* OR home* OR accommodation) N1 shar*)
S64	TI (cohabit* OR co-habit*) OR AB (cohabit* OR co-habit*)
S63	TI (relative OR relatives) OR AB (relative OR relatives)
S62	TI (sibling*) OR AB (sibling*)
S61	TI (child OR children*) OR AB (child OR children*)
S60	TI (son OR sons OR daughter*) OR AB (son OR sons OR daughter*)
S59	TI (family OR families OR familial) OR AB (family OR families OR familial)
S58	TI (household*) OR AB (household*)
S57	TI ((in OR common) N1 (law OR laws)) OR AB ((in OR common) N1 (law OR laws))
S56	TI (grand*) OR AB (grand*)
S55	TI (step-*) OR AB (step-*)
S54	TI (couple OR couples) OR AB (couple OR couples)
S53	TI (significant N1 other*) OR AB (significant N1 other*)
S52	TI (married OR marriage*) OR AB (married OR marriage*)
S51	TI (partner*) OR AB (partner*)
S50	TI (wife OR wives) OR AB (wife OR wives)
S49	TI (husband*) OR AB (husband*)
S48	TI (spous*) OR AB (spous*)
S47	MH "Housing+"
S46	MH "Stepfamilies"
S45	MH "Spouses"
S44	MH "Significant Other"
S43	MH "Siblings"
S42	MH "Paternal Behavior"
S41	MH "Parenting"
S40	MH "Men's Health"
S39	MH "Marriage"
S38	MH "Interpersonal Relations+"

S37	MH "Friendship"
S36	MH "Fathers+"
S35	MH "Father-Infant Relations"
S34	MH "Father-Child Relations"
S33	MH "Fatherhood"
S32	MH "Family+"
S31	MH "Extended Family+"
S30	TI (father*) OR AB (father*)
S29	MH "Expectant Fathers"
S28	S12 OR S13 OR S14 OR S15 OR S16 OR S17 OR S18 OR S19 OR S20 OR S21 OR S22 OR S23 OR S24 OR S25 OR S26 OR S27
S27	TI "expectant mother*" OR AB "expectant mother*"
S26	(MH "Expectant Mothers")
S25	TI (pregnant or pregnanc*) OR AB (pregnant or pregnanc*)
S24	(MH "Breast Feeding")
S23	(MH "Postnatal Period+")
S22	(MH "Infant, Newborn")
S21	(MH "Analgesia, Obstetrical")
S20	(MH "Labor Pain")
S19	(MH "Prenatal Care")
S18	(MH "Perinatal Care")
S17	(MH "Prenatal Diagnosis+")
S16	(MH "Fetal Monitoring+")
S15	(MH "Fetus+")
S14	(MH "Maternal Health Services+")
S13	(MH "Pregnancy Complications+")
S12	(MH "Pregnancy+")
S11	S5 OR S6 OR S7 OR S8 OR S9 OR S10
S10	(MH "Tobacco, Smokeless")
S9	(MH "Tobacco")
S8	(MH "Nicotine")
S7	(MH "Smoking Cessation Programs")
S6	(MH "Smoking")
S5	TI ((stop* or quit* or reduc* or give up or giving up) N2 (cigarette* or tobacco or smoking)) OR AB ((stop* or quit* or reduc* or give up or giving up) N2 (cigarette* or tobacco or smoking))
S4	S1 OR S2 OR S3
S3	qualitative
S2	interview* or (MH "Interviews+")
S1	findings

Social Sciences Citation Index (SSCI)
Searched via Web of Knowledge interface, 09.01.14
Databases=SSCI, CCR-EXPANDED, IC Timespan=1990-2013; Language=(English)

#20	#19 AND #3 AND #2 AND #1
#19	#18 OR #17 OR #16 OR #15 OR #14 OR #13 OR #12 OR #11 OR #10 OR #9 OR #8 OR #7 OR #6 OR #5 OR #4
#18	(TS=((live OR living OR dwell* OR resid* OR stay* OR lodg* OR inhabit*) NEAR/1 (together))) AND Language=(English)
#17	(TS=(guest$ OR visitor$ OR friend$ OR acquaintance$)) AND Language=(English)
#16	(TS=(flatmate$ OR flat-mate$ OR co-renter$ OR corenter$ OR roommate$ OR room-mate$ OR lodger$ OR tenant$ OR landlord$ OR land-lord$)) AND Language=(English)
#15	(TS=((flat$ OR apartment$ OR room$ OR house* OR home$ OR accommodation) NEAR/1 ("same"))) AND Language=(English)
#14	(TS=((flat$ OR apartment$ OR room$ OR house* OR home$ OR accommodation) NEAR/1 (shar*))) AND Language=(English)
#13	(TS=(co-habit* OR cohabit*)) AND Language=(English)
#12	(TS=(family OR families OR familial)) AND Language=(English)
#11	(TS=("significant other" OR "significant others")) AND Language=(English)
#10	(TS=(married OR marriage$)) AND Language=(English)
#9	(TS=(partner$)) AND Language=(English)
#8	(TS=(couple$)) AND Language=(English)
#7	(TS=(husband* OR wife OR wives)) AND Language=(English)
#6	(TS=(spous*)) AND Language=(English)
#5	(TS=(paternal)) AND Language=(English)
#4	(TS=(father*)) AND Language=(English)
#3	(TS=(pregnant or pregnanc* or "expectant mother*")) AND Language=(English)
#2	(TS=(cigarette* or tobacco or smok* or nicotine)) AND Language=(English)
#1	(TS=(qualitativ* or finding* or interview*)) AND Language=(English)

The Economic and Social Research Council (ESRC)
Searched via http://www.esrc.ac.uk/search/advanced-search.aspx, 09.01.14
(The ESRC website's advanced search engine is configured to use the Porter Stemming plugin which reduces search words down to their base word.)

	All of these words:	AND	Any of these words:
1.	qualitative pregnant		cigarette tobacco smoker smoking nicotine
2.	interview pregnant		cigarette tobacco smoker smoking nicotine
3.	finding pregnant		cigarette tobacco smoker smoking nicotine
4.	qualitative pregnancy		cigarette tobacco smoker smoking nicotine
5.	interview pregnancy		cigarette tobacco smoker smoking nicotine
6.	finding pregnancy		cigarette tobacco smoker smoking nicotine
7.	qualitative expectant		cigarette tobacco smoker smoking nicotine
8.	interview expectant		cigarette tobacco smoker smoking nicotine
9.	finding expectant		cigarette tobacco smoker smoking nicotine
10.	qualitative *partner*		cigarette tobacco smoker smoking nicotine
11.	interview *partner*		cigarette tobacco smoker smoking nicotine
12.	finding *partner*		cigarette tobacco smoker smoking nicotine
13.	qualitative *family*		cigarette tobacco smoker smoking nicotine
14.	interview *family*		cigarette tobacco smoker smoking nicotine
15.	finding *family*		cigarette tobacco smoker smoking nicotine
16.	qualitative *household*		cigarette tobacco smoker smoking nicotine
17.	interview *household*		cigarette tobacco smoker smoking nicotine
18.	finding *household*		cigarette tobacco smoker smoking nicotine
19.	qualitative *friend*		cigarette tobacco smoker smoking nicotine
20.	interview *friend*		cigarette tobacco smoker smoking nicotine
21.	finding *friend*		cigarette tobacco smoker smoking nicotine

PubMed (including Medline)
Searched via NCBI interface, 10.01.14
Limited to Ahead of Print Citations and articles published in the last 2 months.

Search	Query
#7	#5 OR #6
#6	#4 AND (2013/11:2014 [edat] OR 2013/11:2014 [crdt] OR 2013/11:2014[dp])
#5	#4 AND pubstatusaheadofprint
#4	#1 AND #2 AND #3
#3	"Pregnant Women"[mesh] OR "Breast Feeding"[mesh] OR "Postpartum Period"[mesh] OR "Infant, Newborn"[mesh] OR "Obstetric Surgical Procedures"[mesh] OR "Analgesia, Obstetrical"[mesh] OR "Labor Pain"[mesh] OR "Prenatal Care"[mesh] OR "Perinatal Care"[mesh] OR "Prenatal Diagnosis"[mesh] OR "Fetal Therapies"[mesh] OR "Fetal Monitoring"[mesh] OR "Fetus"[mesh] OR "Maternal Health Services"[mesh] OR "Pregnancy Complications"[mesh] OR "Pregnancy"[mesh] OR pregnant[tiab] OR pregnanc*[tiab] OR "expectant mother*"[tiab]
#2	"Tobacco Smoke Pollution"[mesh] OR "Tobacco, Smokeless"[mesh] OR "Tobacco"[mesh] OR "Nicotine"[mesh] OR "Tobacco Use Disorder"[mesh] OR "Tobacco Use Cessation"[mesh] OR "Smoking Cessation"[mesh] OR "Smoking"[mesh] OR ((stop*[tiab] OR quit*[tiab] OR reduce*[tiab] OR reduct*[tiab] OR "give up" [tiab] OR "giving up" [tiab]) AND (cigarette*[tiab] OR tobacco[tiab] OR smoking[tiab]))
#1	findings OR qualitative OR interview* OR "Interviews as Topic"[mesh] OR "Interview, Psychological"[mesh]

Google Scholar
Searched via http://scholar.google.co.uk, 10.01.14
Limited to English language pages only and 2013-14 to capture 'ahead of print' but 'published online' articles excluding patents; cookies deleted between searches.

All of these words:	This exact phrase:	Any of these words:
qualitative pregnant	published online	cigarette tobacco smoker smoking nicotine
interview pregnancy	published online	cigarette tobacco smoker smoking nicotine
qualitative "expectant"	published online	cigarette tobacco smoker smoking nicotine
interview "expectant"	published online	cigarette tobacco smoker smoking nicotine
