# Supplementary material for: The barriers and facilitators to smoking cessation experienced by women’s partners during pregnancy and the post-partum period: a systematic review of qualitative research
Source: BMC Public Health. 2015 Sep 3;15:849. doi: 10.1186/s12889-015-2163-x (PMC4558795; doi:10.1186/s12889-015-2163-x)
Supplement: Additional file 2: — Contains details of the included papers, grouped by study. (PDF 288 kb) [file 12889_2015_2163_MOESM2_ESM.pdf]

Additional file 2: Included papers grouped by study (n=9)

Flemming et al (2015). The barriers and facilitators to smoking cessation experienced by women's partners during pregnancy and the post-partum period: a systematic review of qualitative research. *BMC Public Health*. DOI:10.1186/s12889-015-2163-x

Source Paper (n=14)	Country setting	Article title	Participants	Methodology	Indicative finding	Quality Score (out of 36)
Bottorff, Kalaw, Johnson, et al (2006) [19]	Canada	Couple dynamics during women's tobacco reduction in pregnancy and post-partum	28 partners, including one woman recruited from post-natal units. 18 daily smokers, 7 ex-smokers, 4 non-smokers.	Grounded theory.<br><br>Individual interviews at home/by telephone. First at 2-4 weeks post-partum and second at 3-6 months post partum.	Unquestioned expectations for pregnant women's cessation created the social context of compelled tobacco reduction, fundamentally altering couples' previously established tobacco-related routines and were a source of conflict for some couples.	32
Bottorff, Oliffe, Kalaw, et al. (2006) [20]	Canada	Men's constructions of smoking in the context of women's tobacco reduction during pregnancy and post-partum	Secondary analysis of 20 interviews with men self-identified as smokers drawn from main study (above). 18 regular daily smokers (duration 6-21+ years), 2 ex-smokers.	Original study used grounded theory, secondary analysis undertaken using constant comparative methods along with social constructivist perspectives.	Men's reliance on and commitment to dominant ideals of masculinity seemed to preclude them from viewing their partner's tobacco reduction or cessation for pregnancy as an opportunity for cessation.	32
Greaves, Kalaw & Bottorff (2007) [28]	Canada	Case studies of power and control related to tobacco use during pregnancy	Secondary analysis of 3 exemplar cases demonstrating strong examples of tobacco related interaction pattern drawn from main study (above).	Original study used grounded theory, secondary analysis undertaken using the Duluth Domestic Abuse Intervention Power Control Wheel.	Elements of power and control are potentially important and unrecognised dimensions of women's tobacco reduction experiences, causing emotional toll and a negative impact on women's ability to talk about their own tobacco use.	32

**Source Paper (n=14)**	**Country setting**	**Article title**	**Participants**	**Methodology**	**Indicative finding**	**Quality Score (out of 36)**
Bottorff, Radsma, Kelly & Oliffe (2009) [21]	Canada	Father's narratives of reducing and quitting smoking	29 men who had smoked prior to or during their partner's pregnancy. 25 daily smokers and 4 men who quit prior to the birth of their baby. Recruited through post-partum units.	Ethnographic methods.<br><br>Individual interviews at 0-6 weeks & 16-24 weeks post-partum, at home or at a coffee shop by male interviewers.	The different ways men approached reducing and quitting smoking produced four themes: quitting cold turkey, planned reduction; baby displacing the need to smoke; forced reduction.	32
Greaves, Oliffe, Ponic, et al (2010) [26]	Canada	Unclean fathers, responsible men: Smoking, stigma and fatherhood	29 men who had smoked prior to or during their partner's pregnancy.	Ethnographic methods.<br>Individual interviews at 0-6 weeks & 16-24 weeks post-partum, at home or at a coffee shop by male interviewers.	New fathers reported a stigma associated with their position as new fathers who smoked which affected their self-image, smoking behaviour, social reputation and fathering practices.	29
Kwon, Oliffe, Bottorff & Kelly (2014) [24]	Canada	Masculinity and fatherhood: New fathers' perceptions of the female partners efforts to assist them to reduce or quit smoking	Secondary analysis of interviews with 20 new and expectant fathers (drawn from wider study population), all of whom smoked during pregnancy, seven had quit during pregnancy or post-partum, remainder were daily smokers.	Original study used ethnographic methods	Three key themes were identified: support and autonomy<br>in men's smoking cessation, perception of challenging men's freedom to smoke, and contempt for men's continued smoking. These suggest that the design of smoking cessation interventions should consider the shifts in masculinities that occur as men take up fathering	29

Source Paper (n=14)	Country setting	Article title	Participants	Methodology	Indicative finding	Quality Score (out of 36)
Bull, Burke, Walsh & Whitehead (2007) [29]	UK	Social attitudes towards smoking in pregnancy in East Surrey: A qualitative study of smokers, former smokers and non-smokers	5 male partners from a total of 33 women interviewed in a higher & lower income area. All the men were from the lower income area Not all participants smoked (? 2 non-smokers). Recruited through adverts in local newspapers and flyers.	Thematic analysis.<br><br>Individual interviews carried out in participants' homes. Men were interviewed separately from their partners.	Male respondents considered that 'in principle' it was important to quit smoking during pregnancy for medical and social reasons. However it was considered that this was the individual's choice, but that continuing to smoke may invoke social disapproval.	26
Gage (2005) PhD Thesis [16]	USA	Male partnership participation in smoking cessation of women in pregnancy	23 men, 12 interviewed during pregnancy and 11 post-natally.<br>14 were first time fathers. Included 17 current smokers, 4 former smokers and 2 who had never smoked.	Grounded theory with constant comparative analysis.<br><br>Individual interviews in participants' homes.	Pregnancy initially motivated first-time fathers to protect their babies from the negative health effects of cigarette smoking by attempting cessation. In mid-to late pregnancy the preparation and transition to fatherhood caused first-time fathers to consider new roles and responsibilities which altered their perceptions of protection and consequently their smoking goals and behaviours.	*
Gage, Everett & Bullock (2011) [17]	USA	A theoretical explanation of male partner participation in smoking cessation during transition to fatherhood	23 men, 12 interviewed during pregnancy and 11 post-natally.<br>14 were first time fathers. Included 17 current smokers, 4 former smokers and 2 who had never smoked.	Grounded theory with constant comparative analysis.<br><br>Individual interviews in participants' homes.	First time fathers were particularly receptive to quitting smoking early in their partner's pregnancy, but motivation to quit decreased as pregnancy progressed for all men, as the focus shifted from quitting to minimising harm from continued smoking	27

Source Paper (n=14)	Country setting	Article title	Participants	Methodology	Indicative finding	Quality Score (out of 36)
Gould, Munn, Avuri, et al (2013) [22]	Australia	'Nobody smokes in the house if there's a new baby in it'. Aboriginal perspectives on tobacco smoking in pregnancy and in the household in regional NSW Australia	3 male partners/family members (all smokers) along with 15 women, recruited from a maternal and infant health service.	Inductive analysis using constant comparative approach. Data were collected through focus groups.	Smoking in pregnancy provides a sense of social connection, and is seen as acceptable by partners and family members. Risks from smoking were weighed up against personal knowledge. Men tried to maintain smoke free environments in their own homes.	28
Kayser & Semenic (2013) [25]	Canada	Smoking motives, quitting motives and opinions about smoking cessation support among expectant or new fathers	5 expectant fathers & 5 new fathers, all of whom smoked daily and were at the pre-contemplation or contemplation stage of smoking cessation, recruited from the antenatal clinic or post-partum unit.	Descriptive qualitative design using content analysis.<br><br>Individual interviews.	Men made changes to their smoking behaviour to protect their partners & babies from second hand smoke, but were reluctant to quit. Stress associated with pregnancy and childbirth influences continued smoking but there is identified potential for future smoking reduction and cessation.	28
MacLean, Sims-Jones, Hotte & Edwards (2000) [27]	Canada	Perceived partner support and teamwork: Living with tobacco use in childbearing families	8 men partners recruited as part of a couple with their pregnant partner; 3 non-smokers, 1 quit, 1 cut down, 3 continuing smokers, recruited from a variety of courses including ante-natal classes, community health centres.	Descriptive analysis.<br><br>Individual interviews open-ended interviews.	The way in which partners showed support to women through the process of trying to cut down or quit smoking in pregnancy, had important effects on women's ability to quit and stay quit in pregnancy and during the post-partum period.	23

Source Paper (n=14)	Country setting	Article title	Participants	Methodology	Indicative finding	Quality Score (out of 36)
Oliffe, Bottorff, Johnson, et al (2010) [23]	Canada	Fathers: locating smoking and masculinity in the post-partum	20 new fathers who had smoked during their partner's pregnancy. Recruited from ante and post-natal units of a large city hospital. Four had recently quit and 16 were still daily smokers.	Thematic analysis.<br><br>Semi-structured interviews exploring men's experiences of smoking in the context of becoming a new father.<br>Men were also asked to photograph the places they smoked both during and after their partner's pregnancy.<br>A second interview occurred in which the men were invited to discuss each of their photographs.	Smoking adjustments were nestled within the discourse of new fatherhood dedicated to protecting their child and partner from second hand smoke. The photographs and commentaries reveal the highly gendered nature of everyday places where fathers smoke.	32
Wakefield, Reid, Roberts, et al (1998) [12]	Australia	Smoking and smoking cessation among men whose partners are pregnant: a qualitative study	'Four focus group discussions of eight men'. All of the men were smokers. Recruited via ante-natal clinics either directly or through their partners. although only half their pregnant partners smoked.	No report.	Men were mostly unaware that their smoking could affect the health of the foetus; when their partners were smokers, their own smoking seemed unimportant; barriers to quitting included the lack of understanding of the harm caused by SHS lack of motivation to quit in early pregnancy as the baby didn't seem 'real'; concerns about stress within relationships due to cigarette withdrawal.	26

* Quality appraisal not completed for full thesis
